# *catena*-Poly[cadmium-bis­(μ-*N*,*N*-dimethyl­dithio­carbamato-κ^3^ *S*,*S*′:*S*)]

**DOI:** 10.1107/S1600536810043977

**Published:** 2010-10-31

**Authors:** Yue Bing, Xing Li, Meiqin Zha, Yue Lu

**Affiliations:** a Faculty of Materials Science and Chemical Engineering, Ningbo University, Ningbo, Zhejiang 315211, People’s Republic of China

## Abstract

In the title compound, [Cd(C_3_H_6_NS_2_)_2_]_*n*_, the Cd^II^ atom, lying on a twofold rotation axis, is coordinated by six S atoms from four different *N*,*N*-dimethyl­dithio­carbamate ligands in a distorted octa­hedral geometry. The bridging of S atoms of the ligands leads to the formation of a one-dimensional structure along [001].

## Related literature

For general background to metal–organic frameworks, see: Kitagawa *et al.* (2006 ▶); Papaefstathiou & MacGillivray (2003 ▶); Yaghi *et al.* (1998 ▶). For sodium, zinc and copper salts of dimethyl­dithio­carbamate, see: Einstein & Field (1974 ▶); Oskarsson & Ymén (1983 ▶).
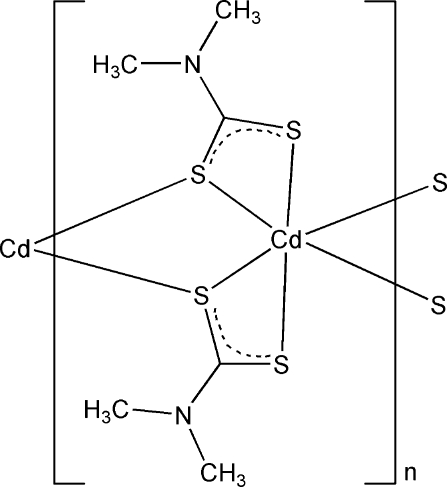


## Experimental

### 

#### Crystal data

[Cd(C_3_H_6_NS_2_)_2_]
*M* *_r_* = 352.82
Orthorhombic, 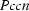

*a* = 10.055 (2) Å
*b* = 14.744 (3) Å
*c* = 7.9518 (17) Å
*V* = 1178.9 (4) Å^3^
*Z* = 4
Mo *K*α radiation
μ = 2.52 mm^−1^
*T* = 296 K
0.54 × 0.22 × 0.17 mm

#### Data collection

Bruker APEXII CCD diffractometer
Absorption correction: multi-scan (*SADABS*; Sheldrick, 1996 ▶) *T*_min_ = 0.519, *T*_max_ = 0.652
9543 measured reflections
1370 independent reflections
1221 reflections with *I* > 2σ(*I*)
*R*_int_ = 0.030

#### Refinement

*R*[*F* ^2^ > 2σ(*F* ^2^)] = 0.017
*wR*(*F* ^2^) = 0.044
*S* = 1.07
1370 reflections
63 parameters
H-atom parameters constrained
Δρ_max_ = 0.29 e Å^−3^
Δρ_min_ = −0.34 e Å^−3^

### 

Data collection: *APEX2* (Bruker, 2007 ▶); cell refinement: *SAINT* (Bruker, 2007 ▶); data reduction: *SAINT*; program(s) used to solve structure: *SHELXS97* (Sheldrick, 2008 ▶); program(s) used to refine structure: *SHELXL97* (Sheldrick, 2008 ▶); molecular graphics: *ORTEP-3* (Farrugia, 1997 ▶); software used to prepare material for publication: *SHELXTL* (Sheldrick, 2008 ▶).

## Supplementary Material

Crystal structure: contains datablocks I, global. DOI: 10.1107/S1600536810043977/hy2367sup1.cif

Structure factors: contains datablocks I. DOI: 10.1107/S1600536810043977/hy2367Isup2.hkl

Additional supplementary materials:  crystallographic information; 3D view; checkCIF report

## Figures and Tables

**Table 1:** Selected bond lengths (Å)

Cd1—S1	2.6255 (7)
Cd1—S2	2.7909 (6)
Cd1—S2^i^	2.7194 (6)

Symmetry code: (i) 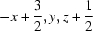.

**Fig. 1.**
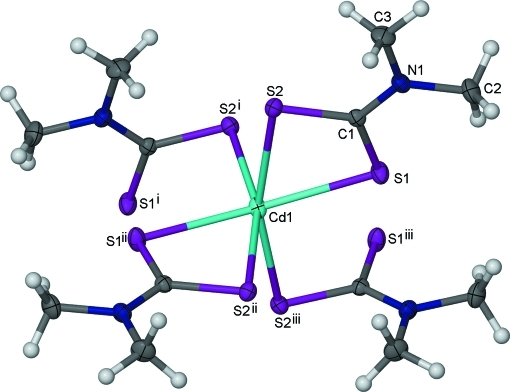
The asymmetric unit of the title compound, showing the Cd coordination. Displacement ellipsoids are drawn at the 30% probability level. [Symmetry codes: (i) 3/2-x, y, 1/2+z; (ii) 3/2-x, 1/2-y, z; (iii) x, 1/2-y, 1/2+z.]

**Fig. 2.**
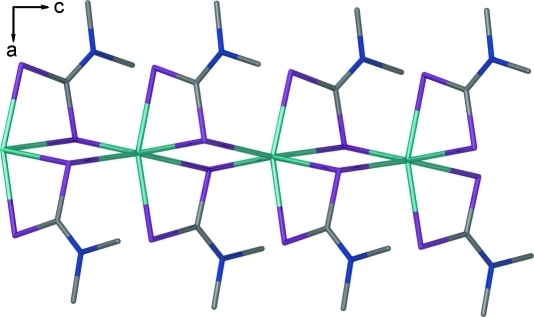
One-dimensional chain in the title complex. H atoms have been omitted for clarity.

## supplementary crystallographic information

### Comment

Rapid development of metal–organic frameworks has been made in recent years not only for their potential applications in materials science but also for fascinating architectures and topologies (Kitagawa *et al.*, 2006; Papaefstathiou & MacGillivray, 2003; Yaghi *et al.*, 1998). Dimethyldithiocarbamic acid is widely used in latex industry. Its natrium, zinc and copper salts are applied widely in antimicrobial, antisepsis and accelerant (Einstein & Field, 1974; Oskarsson & Ymén, 1983). Meanwhile, dimethyldithiocarbamic acid, possessing two S atoms, is a good candidate to coordinate metal atoms and generates rich hydrogen bonding modes. Herein we report the preparation and characterization of the first cadmium complex of dimethyldithiocarbamic acid.

In the title complex, the Cd^II^ ion is coordinated in an octahedral geometry by six S atoms from four different dimethyldithiocarbamate ligands (Fig. 1), with the Cd—S distances ranging from 2.6255 (7) to 2.7909 (6) Å (Table 1). Through the bridging of S2 atoms, the title complex forms a one-dimensional structure (Fig. 2).

### Experimental

A mixture containing 0.005 mmol of Cd(NO_3_)_2_.4H_2_O and 0.010 mmol of dimethyldithiocarbamic acid was placed in a small vial containing MeOH (3.0 ml), DMF (1.0 ml) and H_2_O (0.5 ml). The vial was sealed, heated at 373 K for 2 d and allowed to cool to room temperature. Colorless crystals suitable for X-ray diffraction were collected and dried in air (yield: 50%).

### Refinement

H atoms were placed in calculated positions and treated using a riding model, with C—H = 0.98 Å and with *U*_iso_(H) = 1.5*U*_eq_(C).

### Crystal data

**Table:**

[Cd(C_3_H_6_NS_2_)_2_]	*F*(000) = 696
*M**_r_* = 352.82	*D*_x_ = 1.988 Mg m^−^^3^
Orthorhombic, *P**c**c**n*	Mo *K*α radiation, λ = 0.71073 Å
Hall symbol: -P 2ab 2ac	Cell parameters from 4468 reflections
*a* = 10.055 (2) Å	θ = 2.5–27.6°
*b* = 14.744 (3) Å	µ = 2.52 mm^−^^1^
*c* = 7.9518 (17) Å	*T* = 296 K
*V* = 1178.9 (4) Å^3^	Block, colorless
*Z* = 4	0.54 × 0.22 × 0.17 mm

### Data collection

**Table:**

Bruker APEXII CCD diffractometer	1370 independent reflections
Radiation source: fine-focus sealed tube	1221 reflections with *I* > 2σ(*I*)
graphite	*R*_int_ = 0.030
φ and ω scans	θ_max_ = 27.6°, θ_min_ = 2.5°
Absorption correction: multi-scan (*SADABS*; Sheldrick, 1996)	*h* = −13→13
*T*_min_ = 0.519, *T*_max_ = 0.652	*k* = −16→19
9543 measured reflections	*l* = −10→10

### Refinement

**Table:**

Refinement on *F*^2^	Secondary atom site location: difference Fourier map
Least-squares matrix: full	Hydrogen site location: inferred from neighbouring sites
*R*[*F*^2^ > 2σ(*F*^2^)] = 0.017	H-atom parameters constrained
*wR*(*F*^2^) = 0.044	*w* = 1/[σ^2^(*F*_o_^2^) + (0.0174*P*)^2^ + 0.6307*P*] where *P* = (*F*_o_^2^ + 2*F*_c_^2^)/3
*S* = 1.07	(Δ/σ)_max_ = 0.001
1370 reflections	Δρ_max_ = 0.29 e Å^−^^3^
63 parameters	Δρ_min_ = −0.33 e Å^−^^3^
0 restraints	Extinction correction: *SHELXL97* (Sheldrick, 2008), Fc^*^=kFc[1+0.001xFc^2^λ^3^/sin(2θ)]^-1/4^
Primary atom site location: structure-invariant direct methods	Extinction coefficient: 0.0036 (3)

### Fractional atomic coordinates and isotropic or equivalent isotropic displacement parameters (Å^2^)

**Table:**

	*x*	*y*	*z*	*U*_iso_*/*U*_eq_	
Cd1	0.7500	0.2500	0.17288 (2)	0.03178 (9)	
S1	0.49818 (5)	0.28596 (4)	0.11658 (7)	0.04016 (14)	
S2	0.71139 (5)	0.37665 (3)	−0.08336 (6)	0.03093 (12)	
N1	0.45119 (16)	0.39753 (11)	−0.1396 (2)	0.0335 (4)	
C1	0.54274 (18)	0.35704 (12)	−0.0449 (2)	0.0279 (4)	
C2	0.3088 (2)	0.38157 (18)	−0.1150 (3)	0.0473 (5)	
H2A	0.2952	0.3465	−0.0118	0.071*	
H2B	0.2734	0.3477	−0.2112	0.071*	
H2C	0.2626	0.4399	−0.1057	0.071*	
C3	0.4855 (2)	0.45777 (16)	−0.2802 (3)	0.0481 (5)	
H3A	0.5549	0.5003	−0.2446	0.072*	
H3B	0.4063	0.4917	−0.3151	0.072*	
H3C	0.5181	0.4215	−0.3749	0.072*	

### Atomic displacement parameters (Å^2^)

**Table:**

	*U*^11^	*U*^22^	*U*^33^	*U*^12^	*U*^13^	*U*^23^
Cd1	0.02789 (12)	0.04207 (14)	0.02537 (12)	0.00462 (8)	0.000	0.000
S1	0.0295 (2)	0.0507 (3)	0.0403 (3)	0.0030 (2)	0.0035 (2)	0.0156 (2)
S2	0.0294 (2)	0.0340 (2)	0.0294 (2)	−0.00350 (18)	−0.00062 (18)	−0.00097 (18)
N1	0.0323 (8)	0.0348 (9)	0.0333 (8)	0.0051 (7)	−0.0036 (7)	0.0015 (7)
C1	0.0298 (9)	0.0284 (9)	0.0256 (9)	0.0017 (7)	−0.0001 (7)	−0.0039 (7)
C2	0.0331 (11)	0.0580 (14)	0.0509 (13)	0.0091 (10)	−0.0074 (10)	0.0033 (11)
C3	0.0544 (13)	0.0434 (12)	0.0466 (12)	0.0058 (10)	−0.0070 (11)	0.0154 (10)

### Geometric parameters (Å, °)

**Table:**

Cd1—S1	2.6255 (7)	N1—C3	1.469 (3)
Cd1—S2	2.7909 (6)	C2—H2A	0.9800
Cd1—S2^i^	2.7194 (6)	C2—H2B	0.9800
S1—C1	1.7169 (19)	C2—H2C	0.9800
S2—C1	1.7473 (19)	C3—H3A	0.9800
S2—Cd1^ii^	2.7194 (6)	C3—H3B	0.9800
N1—C1	1.331 (2)	C3—H3C	0.9800
N1—C2	1.464 (3)		
			
S1—Cd1—S1^iii^	160.37 (3)	C1—N1—C2	121.94 (18)
S1—Cd1—S2^i^	96.922 (18)	C1—N1—C3	122.66 (17)
S1^iii^—Cd1—S2^i^	97.039 (16)	C2—N1—C3	115.34 (17)
S1—Cd1—S2^iv^	97.039 (16)	N1—C1—S1	121.09 (14)
S1^iii^—Cd1—S2^iv^	96.922 (18)	N1—C1—S2	119.88 (14)
S2^i^—Cd1—S2^iv^	89.07 (3)	S1—C1—S2	119.03 (10)
S1—Cd1—S2^iii^	98.326 (18)	N1—C2—H2A	109.5
S1^iii^—Cd1—S2^iii^	66.812 (15)	N1—C2—H2B	109.5
S2^i^—Cd1—S2^iii^	163.74 (2)	H2A—C2—H2B	109.5
S2^iv^—Cd1—S2^iii^	94.63 (2)	N1—C2—H2C	109.5
S1—Cd1—S2	66.812 (15)	H2A—C2—H2C	109.5
S1^iii^—Cd1—S2	98.326 (18)	H2B—C2—H2C	109.5
S2^i^—Cd1—S2	94.63 (2)	N1—C3—H3A	109.5
S2^iv^—Cd1—S2	163.74 (2)	N1—C3—H3B	109.5
S2^iii^—Cd1—S2	86.22 (3)	H3A—C3—H3B	109.5
C1—S1—Cd1	89.95 (6)	N1—C3—H3C	109.5
C1—S2—Cd1^ii^	98.61 (6)	H3A—C3—H3C	109.5
C1—S2—Cd1	84.06 (6)	H3B—C3—H3C	109.5
Cd1^ii^—S2—Cd1	92.35 (2)		

Symmetry codes: (i) −*x*+3/2, *y*, *z*+1/2; (ii) −*x*+3/2, *y*, *z*−1/2; (iii) −*x*+3/2, −*y*+1/2, *z*; (iv) *x*, −*y*+1/2, *z*+1/2.
